# Supplementary material for: Volunteering in the Citizen Science Project “Insects of Saxony”—The Larger the Island of Knowledge, the Longer the Bank of Questions
Source: Insects. 2021 Mar 20;12(3):262. doi: 10.3390/insects12030262 (PMC8003976; doi:10.3390/insects12030262)
Supplement: Supplementary file 1 [file insects-12-00262-s001.zip › insects-1146954/Figure S1-S2.pptx]

## Slide 1
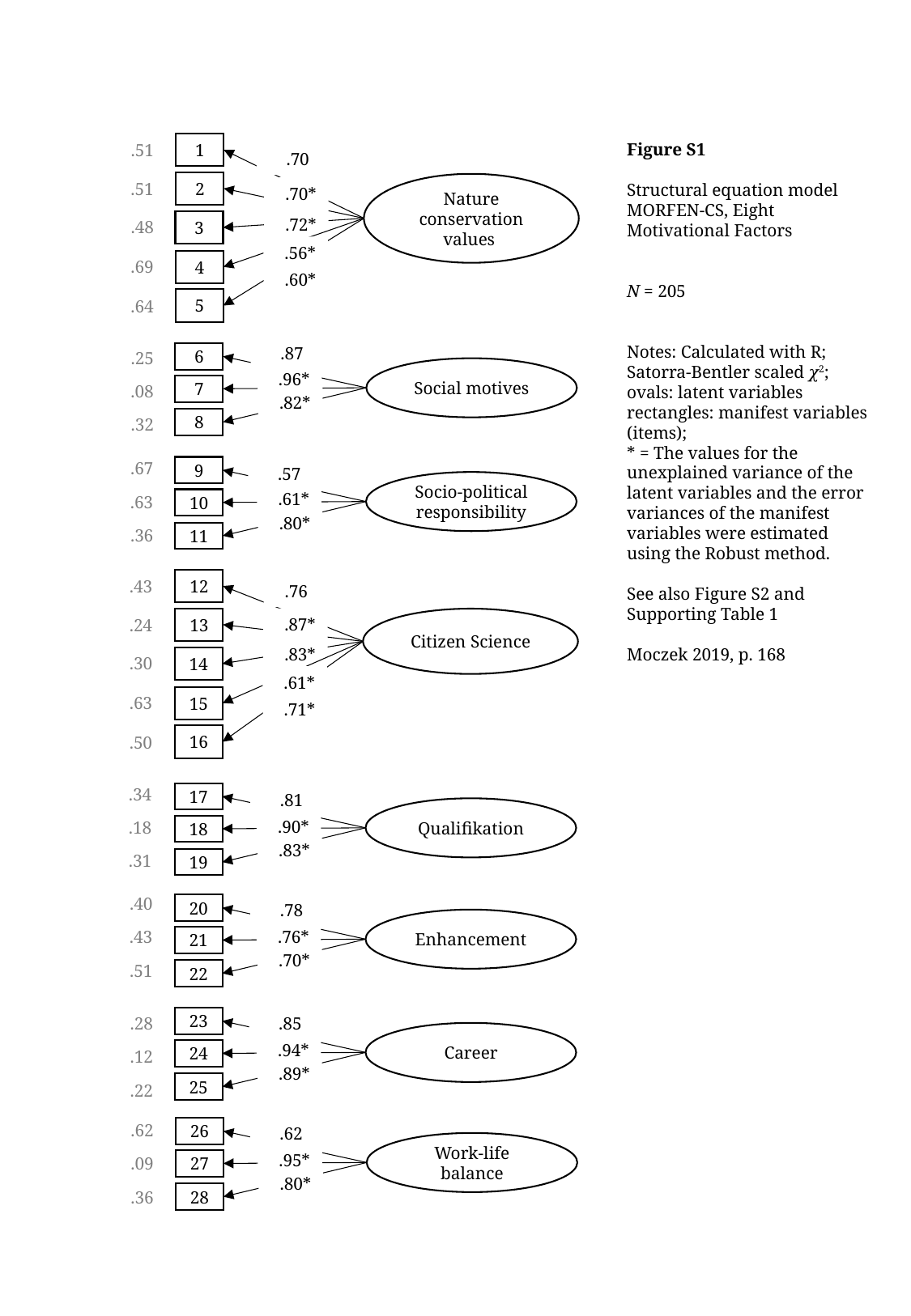

Figure S1
Structural equation model MORFEN-CS, Eight Motivational Factors
N = 205
Notes: Calculated with R; Satorra-Bentler scaled 𝜒2;
ovals: latent variables
rectangles: manifest variables (items);
* = The values for the unexplained variance of the latent variables and the error variances of the manifest variables were estimated using the Robust method.
See also Figure S2 and Supporting Table 1
Moczek 2019, p. 168
1
.51
.70
2
.51
Nature conservation values
.70*
.72*
.48
3
.56*
.69
4
.60*
5
.64
.87
.25
6
Social motives
.96*
.08
7
.82*
.32
8
.67
9
.57
Socio-political responsibility
.61*
.63
10
.80*
.36
11
12
.43
.76*
.87*
13
.24
Citizen Science
.83*
.30
14
.61*
.63
15
.71*
16
.50
.34
17
.81
Qualifikation
.90*
.18
18
.83*
.31
19
.40
.78
20
Enhancement
.43
.76*
21
.70*
.51
22
.28
.85
23
Career
.94*
24
.12
.89*
25
.22
.62
.62
26
Work-life balance
.95*
.09
27
.80*
.36
28

## Slide 2
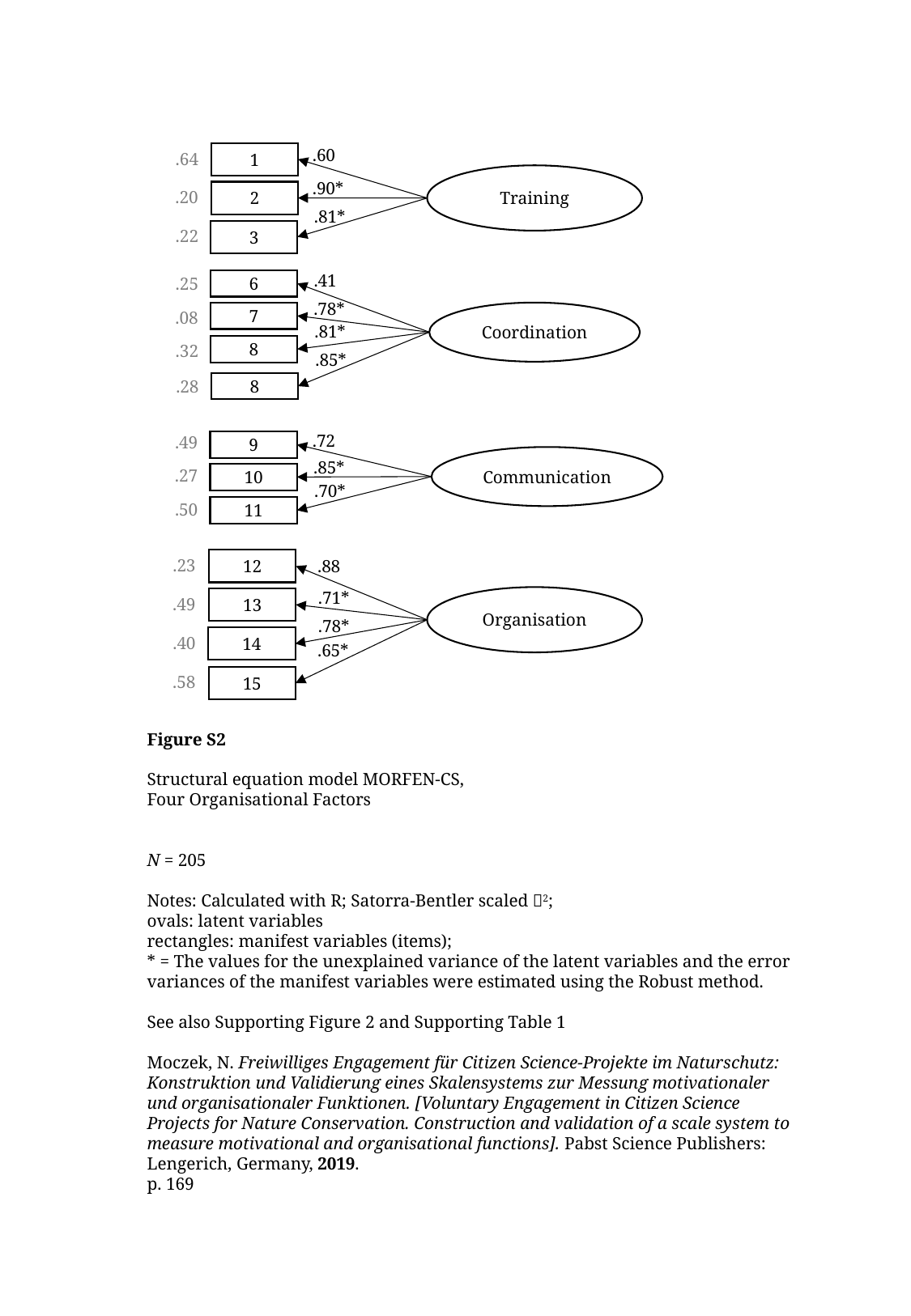

.60*
.64
1
Training
.90*
.20
2
.81*
.22
3
.41*
.25
6
.78*
.08
7
Coordination
.81*
.32
8
.85*
.28
8
.72*
.49
9
Communication
.85*
.27
10
.70*
.50
11
.23
12
.88*
.71*
Organisation
.49
13
.78*
.40
14
.65*
.58
15
Figure S2
Structural equation model MORFEN-CS,
Four Organisational Factors
N = 205
Notes: Calculated with R; Satorra-Bentler scaled 𝜒2;
ovals: latent variables
rectangles: manifest variables (items);
* = The values for the unexplained variance of the latent variables and the error variances of the manifest variables were estimated using the Robust method.
See also Supporting Figure 2 and Supporting Table 1
Moczek, N. Freiwilliges Engagement für Citizen Science-Projekte im Naturschutz: Konstruktion und Validierung eines Skalensystems zur Messung motivationaler und organisationaler Funktionen. [Voluntary Engagement in Citizen Science Projects for Nature Conservation. Construction and validation of a scale system to measure motivational and organisational functions]. Pabst Science Publishers: Lengerich, Germany, 2019.
p. 169
