# Supplementary material for: Volunteering in the Citizen Science Project “Insects of Saxony”—The Larger the Island of Knowledge, the Longer the Bank of Questions
Source: Insects. 2021 Mar 20;12(3):262. doi: 10.3390/insects12030262 (PMC8003976; doi:10.3390/insects12030262)
Supplement: Supplementary file 1 [file insects-12-00262-s001.zip › insects-1146954/Figure S3.docx]

Figure S3: Questionnaire MORFEN-CS, English and German version.

|  | [German] | [English] |
| --- | --- | --- |
|  | Ich engagiere mich in diesem Projekt, weil (ich) ... | I am volunteering in this project, because (I) ... |
|  | **MOTIVATIONALE FUNKTIONEN** | **MOTIVATIONAL FUNCTIONS** |
|  | **Gemeinnützige Motive** | **Pro-social functions** |
|  | **Naturschutzwerte** | **Nature conservation values** |
| 1 | ...etwas für eine Sache tun kann, die mir persönlich wichtig ist. | ...can do something for a cause that is personally important to me. |
| 2 | ...meine persönlichen Werte zu den Projektzielen passen. | ...my personal values match the project goals. |
| 3 | ...in dem Projekt aktiv zum Naturschutz beitragen kann. | ...can actively contribute to nature conservation in the project. |
| 4 | ...mich gerne für (wildlebende) Tiere einsetze. | ...like to support the preservation of wildlife. |
| 5 | ...dem Verlust der Lebensräume etwas entgegensetzen will. | ...want to do something to help to stop the loss of habitats. |
|  | **Soziale Motive** | **Social Motives** |
| 6 | ...dadurch Teil einer Gemeinschaft bin, die sich für dieselbe Sache einsetzt. | ...am part of a community supporting the same cause. |
| 7 | ...mich mit anderen gemeinsam engagieren kann. | ...can get involved together with others. |
| 8 | ...Leute mit ähnlichen Interessen treffe. | ...meet people with similar interests. |
|  | **Gesellschaftspolitische Verantwortung** | **Socio-political responsibility** |
| 9 | ...ich Missstände im Naturschutz beheben helfen möchte. | ...like to rectify deficits in nature conservation. |
| 10 | ...ich eine gesellschaftlich sinnvolle Aufgabe erfüllen möchte. | ...like to perform a socially meaningful task. |
| 11 | ...ich naturschutz-politische Veränderungen anstoßen möchte. | ...like to initiate political changes concerning nature conservation. |
|  | **Citizen Science** | **Citizen Science** |
| 12 | ...damit einem wissenschaftlichen Forschungsprojekt diene. | ...want to support a scientific research project. |
| 13 | ...über das Projekt mit Wissenschaftler*innen in einen fachlichen Austausch komme. | ...am interested in a professional exchange with scientists in the project. |
| 14 | ...wissenschaftliche Prozesse besser verstehen lerne. | ...can learn to understand scientific processes better. |
| 15 | ...damit einen Beitrag zur Artenerfassung und zum Umweltmonitoring leisten kann. | ...want to contribute to species identification and environmental monitoring. |
| 16 | ...zum Austausch des Wissens zwischen Bürger*innen und Forscher*innen beitragen kann. | ...can engage in knowledge exchange among citizens and scientists. |
|  | **Selbstdienliche Motive** | **Self-serving functions** |
|  | **Qualifikation** | **Training** |
| 17 | ...ich neue Sichtweisen auf Natur gewinnen kann. | ...can gain new perspectives on nature. |
| 18 | ...ich durch mein konkretes Handeln Neues lerne und praktisch anwenden kann. | ...through my concrete actions I can learn something new and apply it. |
| 19 | ...ich theoretisches Fachwissen und Methoden lernen und anwenden kann. | ...can learn and apply theoretical knowledge and methods. |
|  | **Anerkennung** | **Enhancement** |
| 20 | ...den Eindruck habe, gebraucht zu werden. | ...get the impression of being needed. |
| 21 | ...für meinen Beitrag eine Anerkennung erhalte. | ...receive recognition for my contribution. |
| 22 | ...mich dabei selbst verwirklichen kann. | ...can self-realise myself. |
|  | **Berufsausgleich** | **Work life balance** |
| 23 | ...in meinem Engagement alles so tun kann, wie ich möchte - anders als im Berufsleben. | ...can do everything I want in my volunteering - unlike in professional life. |
| 24 | ...einen sinnvollen Ausgleich zu meinem Beruf suche. | ...find a meaningful balance to my professional job. |
| 25 | ...mich durch den Aufenthalt in der freien Natur von beruflichen Anforderungen erholen kann. | ...can recover from job requirements by being in nature. |
|  | **Karriere** | **Career** |
| 26 | ...Erfahrungen machen möchte, die ich auch im Beruf nutzen kann. | ...like to make experiences that I can also use in my job. |
| 27 | ...sich das freiwillige Engagement positiv auf meine beruflichen Kompetenzen auswirken kann. | ...volunteering might positively affect my professional skills. |
| 28 | ...Kontakte knüpfen und pflegen kann, die für meine berufliche Entwicklung von Vorteil sein können. | ...can establish and cultivate contacts that can be beneficial for my career. |
|  |  |  |

|  | **ORGANISATIONALE FUNKTIONEN** | **ORGANISATIONAL FUNCTIONS** |
| --- | --- | --- |
|  | Ich engagiere mich in diesem Projekt, weil (ich) ... | I am volunteering in this project, because (I) ... |
|  | **Qualifizierung** | **Qualification** |
| 1 | ...mir klar ist, welche Aufgabe(n) ich im Projekt übernehmen kann. | ...know which tasks I can perform in the project. |
| 2 | ...im Projekt eine Einführung in wissenschaftliche Methoden bekomme. | ...am getting an introduction into scientific methods. |
| 3 | ... mit einer wissenschaftlichen Methode arbeiten kann. | ...can work with scientific methods. |
|  | **Koordination** | **Coordination** |
| 4 | ...Zeit und Dauer meines Engagements selbst bestimmen kann. | ...determine time and duration of my engagement myself. |
| 5 | ...es einen regelmäßigen Austausch mit den Projektkoordinatoren gibt. | ...there is regular contact with the project staff. |
| 6 | ...es im Projekt verschiedene Aufgaben und Aktionen gibt, die ich mir selbst auswählen kann. | ...can choose between different tasks and actions in the project. |
| 7 | ...mich insgesamt gut betreut fühle. | ...am experiencing good support overall. |
|  | **Kommunikation und Rückmeldung** | **Communication and Feedback** |
| 8 | ...zeitnah eine Rückmeldung über die Ergebnisse meiner Arbeit erhalte. | ...promptly get feedback on the results of my work. |
| 9 | ...über die Erfolge im Gesamtprojekt informiert werde. | ...am given information on successes in the overall project. |
| 10 | ...insgesamt den Eindruck habe, dass mein persönliches Engagement hilfreich für das Gesamtprojekt ist. | ...get the impression that my personal engagement is helpful for the entire project. |
|  | **Organisation** | **Organisation** |
| 11 | ...das Projekt insgesamt sehr gut organisiert ist. | ...the project is very well organised overall. |
| 12 | ...Arbeitsmaterialien zur Verfügung gestellt werden. | ...work materials are provided. |
| 13 | ...mir das übergeordnete Projektziel klar ist. | ...the overall project goal is clear to me. |
| 14 | ...das Projekt durch einen Verein/eine Organisation betreut wird. | ...the project is carried by a society/organisation. |

Notes: Six-point scale. 1 = *does not apply at all* ­ 6 = *fully applies*; the bold text was not shown in the survey.

In:

Moczek, N. (2019). *Freiwilliges Engagement für Citizen Science-Projekte im Naturschutz. Konstruktion und Validierung eines Skalensystems zur Messung motivationaler und organisationaler Funktionen [Voluntary Engagement in Citizen Science Projects for Nature Conservation. Construction and validation of a scale system to measure motivational and organisational functions]*. Lengerich: Pabst Science Publishers.
Print: ISBN 978-3-95853-560-2. eBook: ISBN 978-3-95853-561-9.
