# Supplementary material for: Volunteering in the Citizen Science Project “Insects of Saxony”—The Larger the Island of Knowledge, the Longer the Bank of Questions
Source: Insects. 2021 Mar 20;12(3):262. doi: 10.3390/insects12030262 (PMC8003976; doi:10.3390/insects12030262)
Supplement: Supplementary file 1 [file insects-12-00262-s001.zip › insects-1146954/Figure S4.docx]

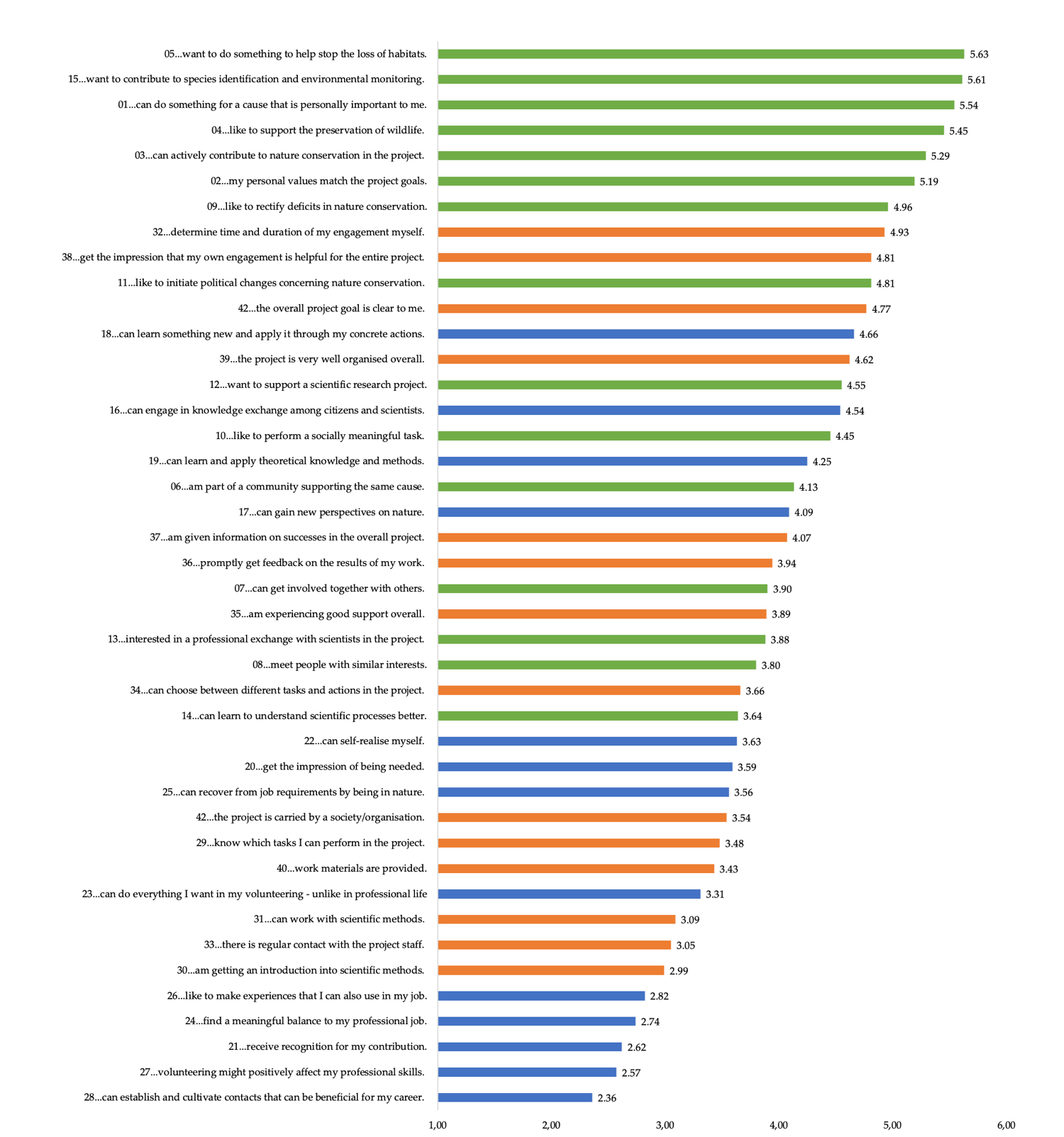


Figure S4: MORFEN-CS, Mean values of items in descending order,
*N* = 116.
Six-point scale, 1 *= does not apply at all ­* 6 *= fully applies*. Items (with numbers) in descending order of expressed consent; green bars = pro-social functions, blue bars = self-serving functions, orange bars = organisational functions.
