# Supplementary material for: Volunteering in the Citizen Science Project “Insects of Saxony”—The Larger the Island of Knowledge, the Longer the Bank of Questions
Source: Insects. 2021 Mar 20;12(3):262. doi: 10.3390/insects12030262 (PMC8003976; doi:10.3390/insects12030262)
Supplement: Supplementary file 1 [file insects-12-00262-s001.zip › insects-1146954/Table S1.docx]

Supporting Table 1

Model comparisons MORFEN-CS (selection)

|  | $\boldsymbol{\chi}$**^2^** | **df** | $\boldsymbol{\chi}$**^2^/df** | **RMSEA** | **SRMR** | **CFI** | **AIC** |
| --- | --- | --- | --- | --- | --- | --- | --- |
| **Motivational functions** |  |  |  |  |  |  |  |
| Model 1:  8-Factors-Model, 28 Items | 539.035** | 322 | 1.67 | .061 | .065 | .925 | 17640 |
| Model 2: 8-Factors-Model, 28 Items,  two factors second order | 624.795** | 341 | 1.83 | .068 | .082 | .901 | 17702 |
| **Organisational functions** |  |  |  |  |  |  |  |
| Model 4: 4-Factors-Model, 14 Items | 135.435** | 71 | 1.91 | .071 | .061 | .954 | 9217 |
| Model 5: 4-Factors-Model, 14 Items,  one factor second order | 145.533** | 73 | 1.99 | .075 | .062 | .948 | 9225 |
| **Motivational and organisational functions** |  |  |  |  |  |  |  |
| Model 6: 12-factor-model, 42 Items | 1244.355** | 753 | 1.65 | .060 | .070 | .896 | 26687 |

Moczek, N. *Freiwilliges Engagement für Citizen Science-Projekte im Naturschutz: Konstruktion und Validierung eines Skalensystems zur Messung motivationaler und organisationaler Funktionen*. [*Voluntary Engagement in Citizen Science Projects for Nature Conservation. Construction and validation of a scale system to measure motivational and organisational functions*]. Pabst Science Publishers: Lengerich, Germany, **2019**.
